# Identification of Candidate Genes for Cold Tolerance at Seedling Stage by GWAS in Rice (*Oryza sativa* L.)

**DOI:** 10.3390/biology13100784

**Published:** 2024-09-30

**Authors:** Huimin Shi, Wenyu Zhang, Huimin Cao, Laiyuan Zhai, Qingxin Song, Jianlong Xu

**Affiliations:** 1 State Key Laboratory of Crop Genetics & Germplasm Enhancement and Utilization, Jiangsu Collaborative Innovation Center for Modern Crop Production, Nanjing Agricultural University, No. 1 Weigang, Nanjing 210095, China; shihm96@163.com; 2 State Key Laboratory of Crop Gene Resources and Breeding, Institute of Crop Sciences, Chinese Academy of Agricultural Sciences, Beijing 100081, China; 13514739633@163.com (W.Z.); chm98316@163.com (H.C.); 3 Shenzhen Branch, Guangdong Laboratory for Lingnan Modern Agriculture, Agricultural Genomics Institute at Shenzhen, Chinese Academy of Agricultural Sciences, Shenzhen 518120, China; zhailaiyuan@caas.cn; 4 National Nanfan Research Institute (Sanya), Chinese Academy of Agricultural Sciences, Sanya 572024, China

**Keywords:** rice, GWAS, cold tolerance, candidate genes, favorable haplotype

## Abstract

**Simple Summary:**

The adverse impact of cold temperatures on rice farming has resulted in a significant decrease in both the yield and quality of rice. Therefore, the identification of quantitative trait loci (QTLs) for cold tolerance (CT) holds immense theoretical and practical value for enhancing CT in rice breeding. In this study, we evaluated CT phenotypes at the seedling stage across 1992 rice germplasms and observed variations in CT among different subpopulations. We successfully identified high CT germplasms within both *geng* and *xian* subspecies. By conducting a genome-wide association study (GWAS), we dissected QTLs associated with CT and discovered favorable haplotypes linked to novel candidate genes at crucial QTLs. These findings offer valuable germplasms exhibiting high CT potential as well as favorable haplotypes that can be utilized for developing new varieties with enhanced CT through marker-assisted selection.

**Abstract:**

Due to global climate change, cold temperatures have significantly impacted rice production, resulting in reduced yield and quality. In this study, we investigated two traits related to the cold tolerance (CT) of 1992 diverse rice accessions at the seedling stage. *Geng* accessions exhibited higher levels of CT compared to *xian* accessions, with the GJ-tmp subgroup displaying the strongest CT. However, extreme CT accessions were also identified within the *xian* subspecies. Through GWAS analysis based on the survival rate (SR) and leaf score of cold tolerance (SCT), a total of 29 QTLs associated with CT at the seedling stage were identified, among which four QTLs (*qSR3.1a*, *qSR4.1a*, *qSR11.1x*, and *qSR12.1a*) were found to be important. Furthermore, five candidate genes (*LOC_Os03g44760*, *LOC_Os04g06900*, *LOC_Os04g07260*, *LOC_Os11g40610*, and *LOC_Os12g10710*) along with their favorable haplotypes were identified through gene function annotation and haplotype analysis. Pyramiding multiple favorable haplotypes resulted in a significant improvement in CT performance. Subsequently, three selected accessions (CX534, B236, and IRIS_313-8565), carrying different superior alleles for CT, were selected and recommended for molecular breeding for CT using marker-assisted selection (MAS). The findings from this study provide valuable resources for enhancing rice’s ability for CT while laying a foundation for the future cloning of novel genes involved in conferring CT.

## 1. Introduction

Rice is one of the most significant food crops, feeding more than half of the world’s population and accounting for 30% of total grain output [1]. However, as the global climate changes, the frequency and intensity of severe temperatures are increasing, and very high and low temperatures have a significant detrimental influence on rice production, resulting in a decline in rice quality and output. China’s rice planting region is vast, and low temperatures will affect practically all rice growing locations to variable degrees. As a result, low temperature has emerged as one of the primary abiotic stresses impacting rice growth and yield in China [2]. Therefore, the discovery of cold tolerance (CT) genes in rice, as well as the identification of signal transduction pathways and regulatory networks in response to low temperature, will provide theoretical guidance and application approaches for the study of the molecular mechanisms of CT and rice genetic improvement.

More than 250 CT QTLs have been found by some domestic and international studies, and the research revealed that the majority of these QTLs came from *japonica* (*geng*) rice [3]. Additionally, more than ninety CT QTLs were identified at the seedling stage, including seventy-one coming from *geng* rice, twenty from *indicate* (*xian*) rice, and seven from wild rice [3]. According to data from the National Rice Data Center (https://www.ricedata.cn/, accessed on 30 August 2024), 113 CT-related genes had currently been cloned, mainly including *CTB4a* [2], *qLTG3-1* [4], *HAN1* [5], *Ctb1* [6], etc. Zhang et al. (2017) [2] investigated a cold-tolerant QTL in NIL1913 and cloned the gene *CTB4a*, which encoded a conserved receptor-like kinase with a rich leucine repeat. Furthermore, the authors further found that *CTB4a* could interact with the β subunit AtpB of ATP synthase to positively regulate the content and activity of ATP under low temperature stress, thereby increasing pollen fertility, improving rice seed setting and yield, and consequently controlling rice tolerance to low temperature stress [2]. *qLTG3-1* was located on chromosome 3 and encoded a protein product composed of 184 amino acids that was localized in the cytoplasm and nucleus [4]. During seed germination, *qLTG3-1* was specifically expressed in the aleurone layer of the seed coat and the epiblast covering the coleoptile, which might improve the germination potential of seeds at low temperatures by regulating the cellular vacuolization of these tissues and thereby inducing the relaxation of these tissues [4]. Mao et al. (2019) [5] used a recombinant inbred line population constructed from rice cultivars 02428 and Teqing to clone a seedling CT QTL *HAN1*, namely *LOC_Os11g29290* on chromosome 11, which encoded an oxidase that could catalyze the conversion of active JA-Il into inactive 12 OH-JA-Ile, and negatively regulated CT in rice. Saito et al. (2001) [7] identified two closely linked CT QTLs *Ctb1* and *Ctb2* related to anther length on chromosome 4 in the cold-resistant rice variety *Norin-PL8*. The *Ctb1* was precisely located and the CT gene *LOC_Os04g52830*, encoding an F-box protein, was identified, which interacted with the E3 ubiquitin ligase component *Skp1* and was implicated in the ubiquitin–proteasome pathway [6,7,8].

A genome-wide association study (GWAS) using single nucleotide polymorphisms (SNPs) in the genome is a method for discovering genetic variants affecting complex traits [9]. CT-associated loci can be quickly located in natural populations by GWAS. For instance, 67 QTLs for the low temperature tolerance of seedlings were identified by GWAS in 295 rice RDP1 varieties, and the correlation between the *OsRYH1* gene and CT was determined [10]. A total of 132 loci, affecting CT, were identified by GWAS using 529 rice materials [11]. Among them, the gene *OsMYB2* related to CY was mapped, and the analysis of the gene haplotype revealed the *xian*-*geng* differentiation of this gene [11]. By GWAS, 53 QTLs related to seed germination under low temperatures were identified using a natural rice population of 187 accessions [12]. Researchers discovered that *OsSAP16* was a potential gene influencing cold seed germination. *OsSAP16* function loss decreased cold germination, but *OsSAP16* over-expression increased low-temperature germination [12]. A large number of QTLs related to CT were discovered by GWAS, and there were a lot of candidate genes identified.

Based on DNA diversity, rice can be classified into 12 ecological types, including *GJ*-adm, *GJ*-subtropical, *GJ*-temperate, *GJ*-tropical, XI-1A, XI-1B, XI-2, XI-3, XI-adm, *aus*, *basmati*, and *admix* [13,14,15]. Overall, the *geng* (GJ) rice subspecies, particularly *GJ*-temperate rice, displayed excellent CT while the *xian* (XI) rice subspecies was sensitive to low temperatures [5,16,17]. In this research, GWAS was conducted for the CT-related traits using 1992 rice germplasm accessions (including 473 *geng* and 1290 *xian*) from the 3K Rice Genome Project (3K-RGP). This study clearly aimed to explore the genomic regions and candidate genes related to CT in the whole rice genome and screen breeding materials that could be used for molecular marker-assisted selection (MAS). Our results may facilitate rice breeding for CT to overcome the adverse effects of low temperature on rice yield.

## 2. Materials and Methods

### 2.1. Plant Materials

A total of 1992 accessions were chosen for this research from the 3K-RGP [15], including 473 *geng*, 1290 *xian*, 61 *admix*, 133 *aus*, and 35 *basmati* (Table S1). The *xian* subgroups were further divided into XI-1, XI-2, XI-3, and XI-adm. The *geng* subgroups were additionally categorized as GJ-trp, GJ-sbtrp, GJ-adm, and GJ-tmp [15]. Among them, the origin of XI-1 can be traced back to East Asia; XI-2 is mainly found in West Asia and South Asia; XI-3 derives from Southeast Asia; GJ-trp originates from Southeast Asian island countries; GJ-sbtrp originates from Southeast Asia; GJ-adm and GJ-tmp mainly have their origins in China and Europe, respectively; XI-adm mainly derives from China and India; and *Aus* mainly originates from Bangladesh and India whereas *Basmati* primarily comes from India (Table S1).

### 2.2. Phenotypic Evaluation

The test materials were placed in an air oven set at 50 °C for 72 h to break dormancy, then soaked for 48 h at 37 °C, germinated for 24 h at 37 °C, and finally seeded in a seedling tray (60 cm length × 30 cm width × 5 cm height) filled with universal nutritious soil consisting of peat, perlite, leaf mold, and vermiculite in the proportions of 50% peat, 25% perlite, 15% leaf mold, and 10% vermiculite, with 21 accessions per seedling tray. Two replicates were set for the experiment, and the seeds were cultivated in the phytotron (13 h light at 28 °C and 11 h of darkness at 26 °C) until the seedlings were at the three-leaf stage. After removing weak seedlings, cold treatment was applied at 9 °C in an artificial climate chamber for 5 days based on the preliminary testing, then moved out to recover in the phytotron for 7 days. Then, the survival rate (SR) was calculated as: survival rate (%) = No. of surviving plants/No. of plants × 100. The leaf score of cold tolerance (SCT) was recorded as follows: 1: only the tip of a leaf is yellow or no leaf damage; 3: one-third of the leaves on the third leaf are yellow or withered; 5: roughly two-thirds of the leaves on the second and third leaves are yellow and withered; 7: the second leaf is completely withered, the third leaf is mostly withered but the center is still intact; and 9: the plant is completely dead and all of the leaves are wilted.

### 2.3. Genome-Wide Association Mapping

The SNP genotyping information of 1992 samples was extracted from the 4.8 M SNP dataset of the 3K-RGP [18] using PLINK software (version 1.9) [19]. SNPs with a missing rate > 20% and minor allele frequencies (MAF) < 5% were filtered. The association analysis between the SNPs and CT was performed using a mixed linear model (MLM) in EMMAX (version emmax-beta-07Mar2010) [20]. The filtered SNPs were used to calculate the kinship matrix and principal components based on GCTA software (version v1.94.1 Linux), and the first three principal components as covariates to control the population structure [21]. The effective numbers of SNPs (N) were estimated utilizing GEC software (Version 0.2) [22]. The significant threshold (1/N) of the suggestive *p*-value was then determined using the Bonferroni correction method, setting the whole population at (*p* = 1.97 × 10^−6^), the *geng* subpopulation at (*p* = 5.27 × 10^−6^), and the *xian* subpopulation at (*p* = 2.72 × 10^−6^), respectively. The “qqman” package of the R software (version 4.1.0) was used to create the Manhattan and Q-Q plots [22]. We calculate the linkage distance (LD) of 1992 samples using PopLDdecay software (version 3.42) [23]. The lead SNP in a locus was defined as the SNP with the lowest value of *p*, and the other significant SNPs within 150 kb on either side of the lead SNP were merged as a single association locus (Figure S1), which was consistent with the reported genome-wide linkage disequilibrium (LD) decay in 3K-RGP [15].

### 2.4. Haplotype Analysis for Candidate Genes

We screened potential candidate genes for cold tolerance when they met at least one of the following criteria: (1) We would match the genes with extremely significant as-sociation SNPs (*p*-value < 1/N) in each characteristic (the downstream genes are selected if the SNP is located between the genes) as important candidate genes; (2) The funRiceGenes database [24] and the Nipponbare reference genome IRGSP 1.0 [25] were used to locate genes with abiotic stress-related functional annotations. The haplotype analysis was performed on all candidate genes using all SNPs within the gene coding sequence region [26]. Duncan’s multiple range post-hoc tests were used to compare phenotypic differences between haplotypes (*n* ≥ 30 rice accessions).

## 3. Results

### 3.1. Phenotypic Variations in the Cold Tolerance (CT) of Rice at the Seedling Stage

**Figure 1:**
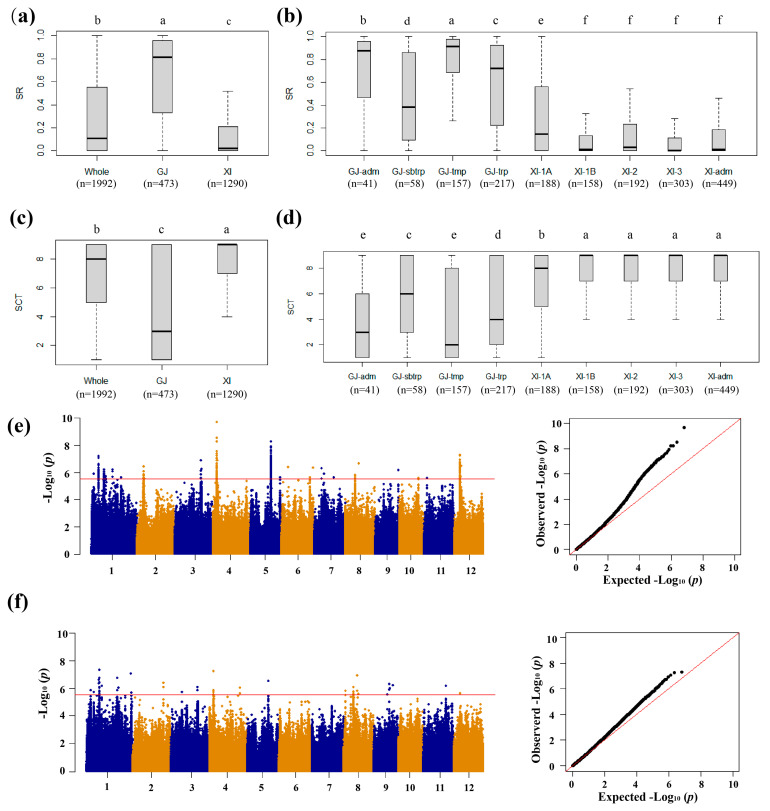
Phenotypic variations in cold tolerance and identification of QTLs affecting cold tolerance through GWAS analysis of rice accessions from the 3K-RGP. (**a**) Box-plots of survival rate (SR) for the whole population, and *xian* (XI) and *geng* (GJ) subpopulations. (**b**) Box-plots of SR among *GJ-adm*, GJ-subtropical (*GJ-sbtrp*), GJ-temperate (*GJ-tmp*), GJ-tropical (*GJ-trp*), XI-1A, XI-1B, XI-2, XI-3, and XI-adm accessions. (**c**) Box-plots of SCT for the whole population, and XI and GJ subpopulations. (**d**) Box-plots of SCT among GJ-adm, GJ-sbtrp, GJ-tmp, GJ-trp, XI-1A, XI-1B, XI-2, XI-3, and XI-adm accessions. (**e**) Manhattan and Q-Q plots of GWAS results for SR. (**f**) Manhattan and Q-Q plots of GWAS results for SCT. Horizontal lines indicate in the Manhattan plots indicate the genomewide suggestive thresholds. In (**a**–**d**), different letters indicate significant differences (*p* < 0.05, Duncan’s multiple range posthoc test).

Two traits including SR and SCT were measured for 1992 accessions, including 473 *geng* and 1290 *xian* accessions at the seedling stage (Table S1). We found that the *geng* subpopulation had significantly higher cold tolerance than *xian* (Figure 1a,c). We observed that fifty-one accessions had an SR of 100%, including five *xian* accessions and forty-six *geng* accessions. A total of six hundred thirteen accessions had an SR of zero, including five hundred fifty-seven *xian* accessions, thirty *geng* accessions, seven *admix* accessions, ten *aus* accessions and nine *bas* accessions (Table S1). In addition, we found some accessions exhibited extreme phenotypes in the SCT trait, and one hundred thirty-six accessions had no leaf damage in the CT test, including eleven *xian* accessions, one hundred seventeen *geng* accessions, four *admix* accessions, two *aus* accessions, and two *bas* accessions (Table S1), indicating that rice germplasms had wide differentiation in CT among different subpopulations.

There was obvious differentiation in CT even in the same subspecies. The mean SR in the *geng* subpopulation was 64.58%, ranging from 0 to 100% (Figure S2c). Similarly, its mean SCT was 4.56, with a range of 1.0 to 9.0 (Figure S1d). Among the four *geng* subgroups, *GJ-tmp* was significantly higher than the others, whereas most accessions with cold-sensitivity belonged to *GJ-sbtrp* (Figure 1b,d). The mean SR in the *xian* subpopulation was 15.54%, ranging from 0 to 100%, and the mean SCT was 7.67, with a range of 1.0 to 9.0 (Figure S2e,f). Among the five *xian* subgroups, XI-1A was significantly more resistant to cold than the others, indicating that subgroup XI-1A had stronger CT than other *xian* subgroups.

According to the correlation analysis among the CT traits in the whole population, SR was significantly negatively correlated with SCT (*r* = −0.84). In addition, correlations of CT-related traits in the subpopulations *xian* and *geng* were similar to that in the whole population.

### 3.2. GWAS for CT

**Figure 2:**
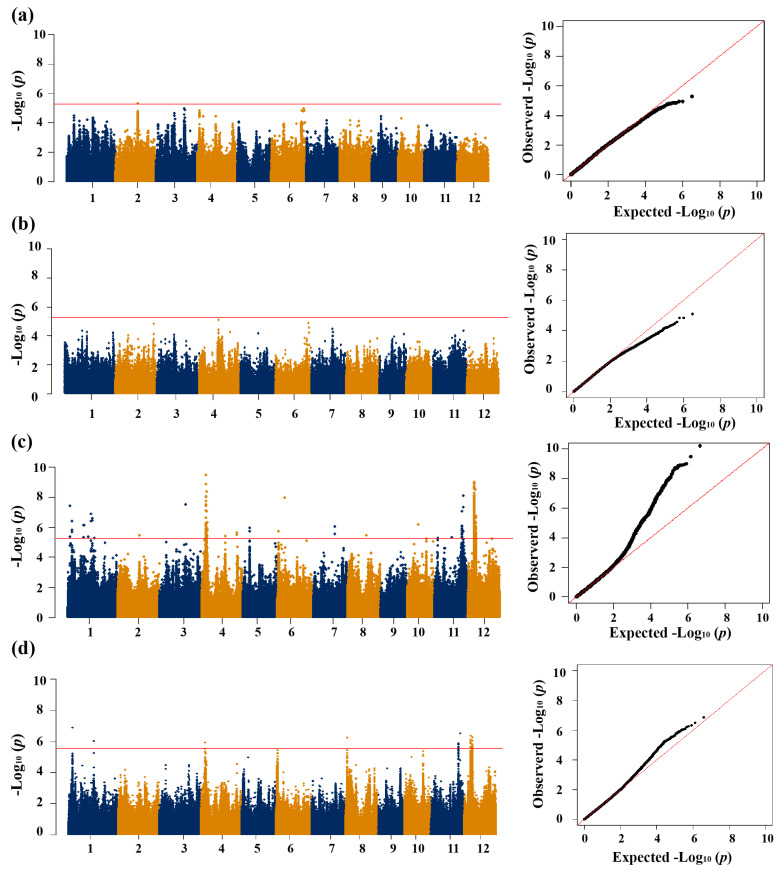
Genome-wide association study of cold tolerance-related traits in the GJ and XI subpopulations. (**a**) SR in the GJ subpopulation. (**b**) SCT in the GJ subpopulation. (**c**) SR in the XI subpopulation. (**d**) SCT in the XI subpopulation. In (**a**–**d**), the horizontal red lines represent the suggestive significant threshold. Horizontal lines indicate in the Manhattan plots indicate the genome-wide suggestive thresholds.

We conducted GWAS based on the mixed linear model for the traits related to CT (Figure 1e,f and Figure 2). Using a Bonferroni correction based on the effective numbers of SNPs, the genome-wide significant values of *p* thresholds were set at 1.97 × 10^−6^, 5.27 × 10^−6^ and 2.72 × 10^−6^ for the whole population, *geng* subpopulation, and *xian* subpopulation, respectively. A total of 16, 1, and 12 QTLs significantly associated with CT at the seedling stage were identified in the whole population, *geng* subpopulation, and *xian* subpopulation, respectively (Table 1 and Table 2).

In the whole population, a total of sixteen QTLs for two traits (SR and SCT) were identified, including eight for SR and eight for SCT (Table 1). Among them, *qSR1.2a* for SR and *qSCT1.1a* for SCT were mapped together in the region of 11.71–11.97 Mb on chromosome 1. The QTLs *qSR3.1a* and *qSCT3.1a* were simultaneously identified in the region of 25.11–25.41 Mb on chromosome 3. The QTLs *qSR4.1a* and *qSCT4.1a* were identified together in the region of 3.58–3.78 Mb on chromosome 4.

A total of one QTL was detected in the *geng* subpopulation on chromosomes 2, including *qSR2.1g* (Table 2). The QTL *qSR2.1g* was detected in the region of 19.64–19.94 Mb on chromosome 2, and affected the SR.

For the *xian* subpopulation, a total of twelve QTLs affecting CT-related traits were mapped on chromosomes 1, 4, 5, 8, 11, and 12, including six QTLs for SR and six for SCT (Table 2). Among them, *qSR1.1x* and *qSCT1.1x*, *qSR4.1x* and *qSCT4.1x*, *qSR11.1x* and *qSCT11.1x*, and *qSR12.1x* and *qSCT12.1x* were commonly detected in the regions of 3.45–3.75 Mb on chromosome 1, 3.63–4.23 Mb on chromosome 4, 24.03–24.33 Mb on chromosome 11, and 5.71–7.25 Mb on chromosome 12, respectively.

Among the 29 QTLs detected for the two traits through GWAS in the three populations, four QTLs (*qSR4.1a*, *qSCT4.1a*, *qSR4.1x*, and *qSCT4.1x*) were co-identified in both the whole and *xian* populations, while two QTLs (*qSR12.1a* and *qSR12.1x*) were found exclusively in these two populations as well. However, no co-detected QTLs were observed between the whole and *geng* populations, nor were any QTLs detected simultaneously within the *geng* and *xian* subpopulations. These findings suggest a significant allele differentiation in CT between the *xian* and *geng* subspecies.

QTLs simultaneously detected for whole and *xian* populations could be used as important QTLs for excavating candidate genes associated with rice CT. Six important QTLs (*qSR1.2a*, *qSR3.1a*, *qSR4.1a*, *qSR1.1x*, *qSR11.1x*, and *qSR12.1a*) were found in different traits for the whole and *xian* populations. Among them, *qSR1.2a* and *qSR1.1x* coincided with the previously cloned genes *OsLEA9* and *OsPLDα1* for CT in rice, respectively [27,29]. So, *qSR3.1a*, *qSR4.1a*, *qSR11.1x*, and *qSR12.1a* were considered as newly identified QTLs for rice CT in this study, and thus were used for further candidate gene analysis.

### 3.3. Haplotype Analyses of the Candidate Genes

**Figure 3:**
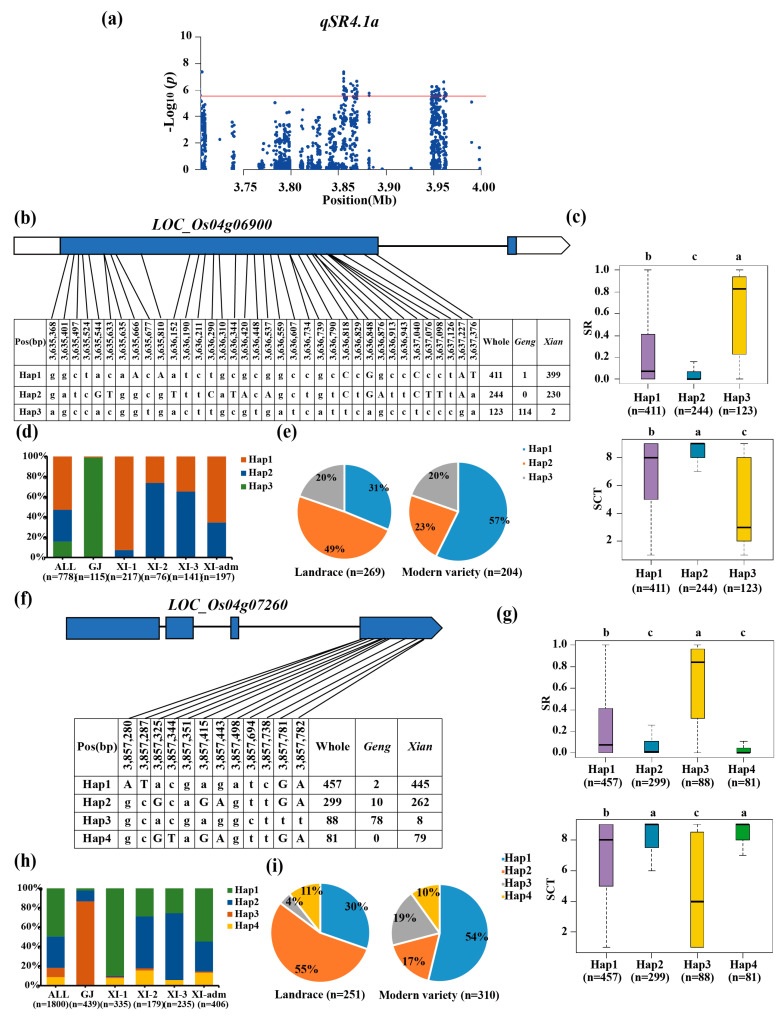
Candidate gene analysis of *qSR4.1a* for cold tolerance. (**a**) Local Manhattan plot (top) of 150 kb upstream and downstream around the lead SNP rs4_3,633,378 (*LOC_Os04g06900*) and rs4_3,855,187 (*LOC_Os04g07260*). Codon-haplotypes of *LOC_Os04g06900* (**b**) and *LOC_Os04g07260* (**f**). The distribution of SR in the accessions for haplotypes (n > 40 accessions) of *LOC_Os04g06900* (**c**) and *LOC_Os04g07260* (**g**). Different letters above each boxplot indicate significant differences among haplotypes (*p* < 0.05, Duncan’s multiple range post-hoc test). Haplotype frequency distribution of *LOC_Os04g06900* (**d**) and *LOC_Os04g07260* (**h**) in different subpopulations. The type of each accession was from the metadata of the 3K-RGP [15]. Frequency of haplotypes of *LOC_Os04g06900* I and *LOC_Os04g07260* (**i**) in the landrace and modern variety populations. Letter n indicates the number of rice accessions belonging to the corresponding subpopulations in (**c**,**d**,**g**,**h**), or the variety type in (**e**,**i**), respectively. Horizontal lines indicate in the Manhattan plots indicate the genome-wide suggestive thresholds.

Two candidate genes, *LOC_Os04g06900* and *LOC_Os04g07260*, were identified using haplotype analysis and gene function analysis for *qSR4.1a/qSCT4.1a/qSR4.1x/qSCT4.1x*. Haplotype analysis indicated that *LOC_Os04g06900* has three major haplotypes; Hap3 was determined as the favorable haplotype with the significantly highest SR (Figure 3b,c). This haplotype was enriched in *geng* subpopulation (92.68%) accessions, with an average SR of 63% (Figure 3c,d). Hap1 and Hap2, as cold-sensitive haplotypes, were significantly enriched for the *xian* subgroup with SR values of 24% and 9%, respectively (Figure 3d). Furthermore, the proportion of the whole set of accessions with *LOC_Os04g06900*^Hap3^ was no different between the landrace and modern varieties (Figure 3e). Candidate gene *LOC_Os04g07260* was identified as having four major haplotypes (Figure 3f). Among them, *LOC_Os04g07260*^Hap3^, with the significantly highest SR (65%), was considered as the favorable haplotype, which was significantly enriched in the *geng* subpopulation (86.67%) (Figure 3g,h). *LOC_Os04g07260*^Hap2^ and *LOC_Os04g07260*^Hap4^, as cold-sensitive haplotypes, were enriched in the *xian* subpopulation with SR values of 23% and 11%, respectively (Figure 3h). The fraction of the whole set of accessions containing *LOC_Os04g07260*^Hap3^ increased from 4% in the landrace to 19% in the modern variety (Figure 3i).

**Figure 4:**
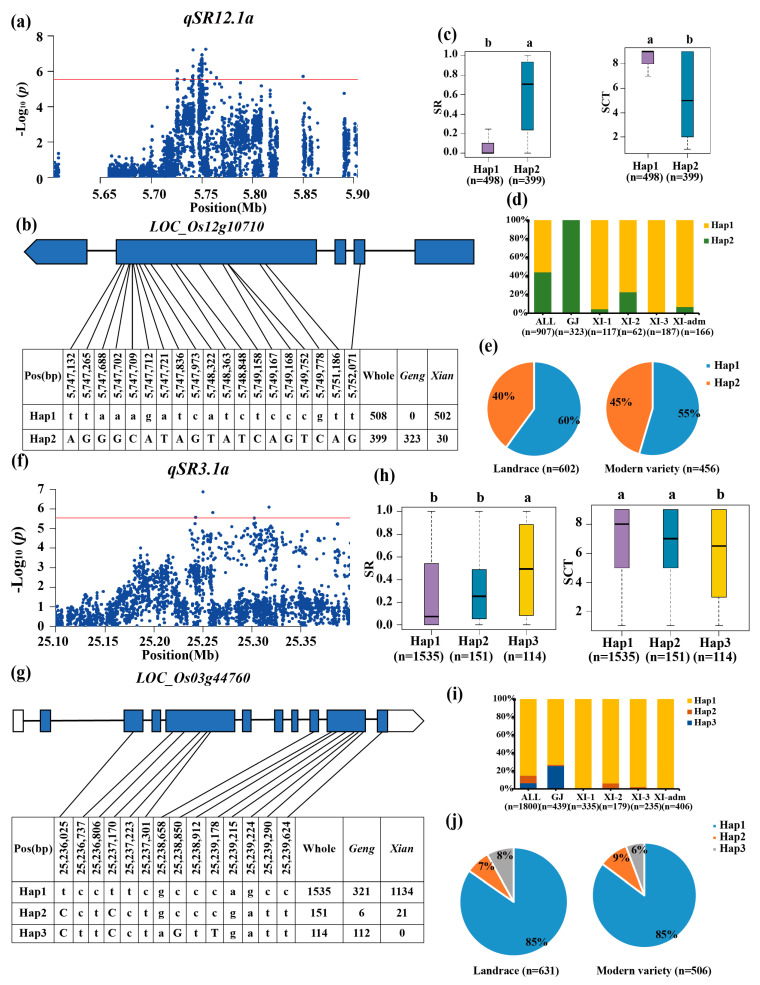
Candidate gene analysis of *qSR12.1a* and *qSR3.1a* for cold tolerance. Local Manhattan plot (top) of 150 kb upstream and downstream around the lead SNP rs12_5,753,724 (*LOC_Os12g10710*) (**a**) and rs3_25,249,852 (*LOC_Os03g44760*) (**f**). CDS-haplotypes of *LOC_Os12g10710* (**b**) and *LOC_Os03g44760* (**g**). The distribution of SR in the accessions for haplotypes (n > 40 accessions) of *LOC_Os12g10710* (**c**) and *LOC_Os03g44760* (**h**). Different letters above each boxplot indicate significant differences among haplotypes (*p* < 0.05, Duncan’s multiple range post-hoc test). Haplotype frequency distribution of *LOC_Os12g10710* (**d**) and *LOC_Os03g44760* (**i**) in different subpopulations. The type of each accession was from the metadata of the 3K-RGP [15]. Frequency of haplotypes of *LOC_Os12g10710* (**e**) and *LOC_Os03g44760* (**j**) in the landrace and modern variety populations. Letter n indicates the number of rice accessions belonging to the corresponding subpopulation in (**c**,**d**,**h**,**i**), or the variety type in (**e**,**j**), respectively. Horizontal lines indicate in the Manhattan plots indicate the genome-wide suggestive thresholds.

Candidate gene *LOC_Os12g10710* was identified at *qSR12.1a/qSR12.1x/qSCT12.1x* (Figure 4a). *LOC_Os12g10710* was recognized as having two major haplotypes (Figure 4b). The favorable haplotype was identified to be Hap2 with the highest SR (59%) and was significantly enriched in the *geng* subpopulation accessions (Figure 4c,d). Hap1, as a cold-sensitive haplotype, was significantly enriched for the subgroup *xian* with SR values of 9% (Figure 4c,d). Furthermore, the fraction of the whole set of accessions containing *LOC_Os12g10710*^Hap2^ increased from 40% in the landrace to 45% in the modern variety (Figure 4e).

For locus *qSR3.1a/qSCT3.1a* on chromosome 3, the candidate gene *LOC_Os03g44760* was predicted by haplotype analysis and gene function analysis. *LOC_Os03g44760* had a lead SNP rs3_25,249,852 (*p* = 1.36 × 10^−7^) for SR in the whole population (Figure 4f–h). Three major haplotypes were identified for *LOC_Os03g44760* (Figure 4h), and Hap3 with the significantly highest SR (49%) was determined as the favorable haplotype, which was significantly enriched in the *geng* subpopulation (Figure 4i). Furthermore, the proportion of the whole set of accessions with *LOC_Os03g44760*^Hap3^ dropped from 8% in landrace to 6% in the modern variety (Figure 4j).

**Figure 5:**
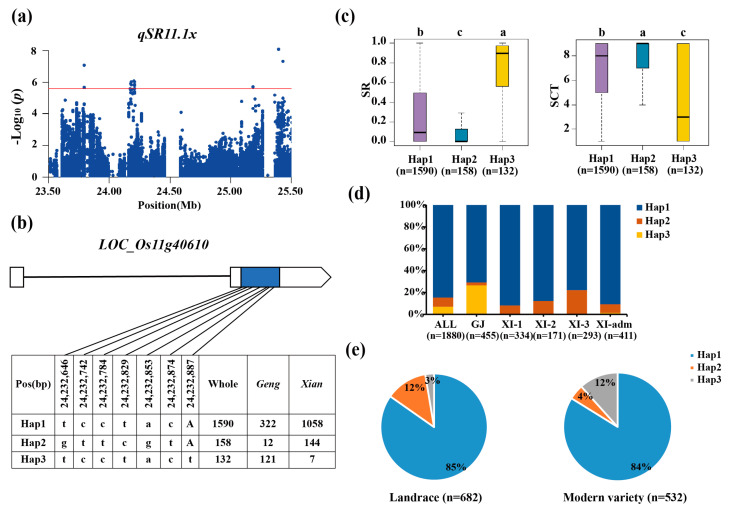
Candidate gene analysis of *qSR11.1x* for cold tolerance. (**a**) Local Manhattan plot (top) of 150 kb upstream and downstream. (**b**) CDS-haplotypes of *LOC_Os11g40610*. (**c**) The distribution of SR in the accessions for haplotypes (n > 40 accessions) of *LOC_Os11g40610*. Different letters above each boxplot indicate significant differences among haplotypes (*p* < 0.05, Duncan’s multiple range post-hoc test). (**d**) Haplotype frequency distribution of *LOC_Os11g40610* in different subpopulations. The type of each accession was from the metadata of the 3K-RGP [15]. (**e**) Frequency of haplotypes of *LOC_Os11g40610* in the landrace and modern variety of the 3K-RGP. Letter n indicates the number of rice accessions belonging to the corresponding subpopulation in (**c**,**d**), or the variety type in (**e**), respectively. Horizontal lines indicate in the Manhattan plots indicate the genome-wide suggestive thresholds.

The candidate gene *LOC_Os11g40610* at *qSR11.1x/qSCT11.1x* on chromosome 11 (Figure 5a) was shown to be linked to stress tolerance, indicating that it is a relevant candidate gene (Figure 5b) [30]. The favorable haplotype among the three major haplotypes was found to be Hap3, which had the highest SR (74%) (Figure 5c). For the whole population, the haplotype *LOC_Os11g40610*^Hap3^ was highly enriched in the *geng* subpopulation accessions (Figure 5d). The *LOC_Os11g40610*^Hap3^ increased from 3% in the landrace to 12% in the modern variety (Figure 5e).

### 3.4. Optimal Combination of CT-Haplotypes

**Figure 6:**
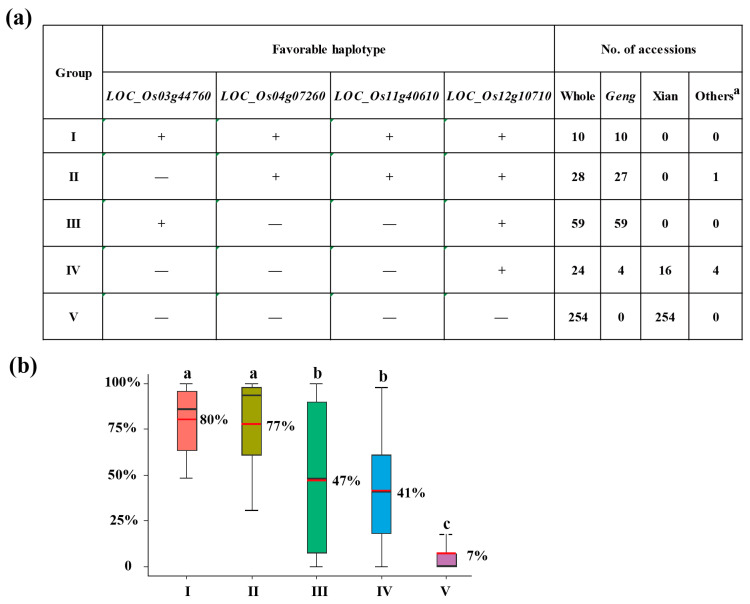
Optimal combinations of four favorable haplotypes for cold tolerance. (**a**) Five combinations of four favorable haplotypes at *LOC_Os04g07260*, *LOC_Os12g10710*, *LOC_Os03g44760*, and *LOC_Os11g40610*, and the distribution patterns of these accessions across different subpopulations. “+” and “−” represent favorable and inferior haplotypes, respectively. ^a^ means other subpopulations except *geng* and *xian* subspectASies. (**b**) Comparisons of the SR among accessions with different haplotype combinations. Different letters above each histogram indicate significant differences at *p* < 0.05 (least significant difference test).

Since SR is a key trait for CT, it was used to identify the favorable haplotype of each candidate gene. Based on the haplotype analysis mentioned above, *LOC_Os04g06900*^Hap3^, *LOC_Os04g07260*^Hap3^, *LOC_Os12g10710*^Hap2^, *LOC_Os03g44760*^Hap3^, and *LOC_Os11g40610*^Hap3^ were identified as favorable CT-haplotypes. The pyramiding effects of different haplotypes on CT were analyzed. There remained five groups comprising four candidate genes after the removal of rare haplotype combinations (n ≥ 10 accessions). The groups I, II, and III were enriched in the *geng* subpopulation, whereas groups IV and V were mainly found in the *xian* subpopulation, which showed clear *xian–geng* differentiation in CT (Figure 6a). Compared to the other four groups, Group I exhibited the highest SR (80%) among all of them, as it contained the cold-tolerant haplotypes at *LOC_Os04g07260*, *LOC_Os12g10710*, *LOC_Os03g44760*, and *LOC_Os11g40610* (Figure 6b). Group II also showed a relatively high SR (77%), possessing the cold-tolerant haplotypes at *LOC_Os04g07260*, *LOC_Os12g10710*, and *LOC_Os11g40610* (Figure 6b). Both groups I and II had significantly higher SR than those groups III and IV which carried cold-tolerant haplotypes at two genes (*LOC_Os03g44760* and *LOC_Os12g10710*) and one gene (*LOC_Os12g10710*), respectively (Figure 6b). These findings suggest that pyramiding more favorable haplotypes of these candidate genes enhances CT. After screening for the favorable cold tolerant haplotypes of *LOC_Os04g07260*^Hap3^, *LOC_Os12g10710*^Hap2^, *LOC_Os03g44760*^Hap3^, and *LOC_Os11g40610*^Hap3^ in 3K-RGP materials, accessions with four favorable haplotypes (CX534) and three favorable haplotypes (B236 and IRIS_313-8565) were obtained. Furthermore, these results indicate that it is an effective strategy to improve rice CT at the seedling stage by pyramiding multiple favorable haplotypes/alleles.

## 4. Discussion

Rice is particularly vulnerable to low temperature stress, and the impact of low temperature on rice yield is sometimes severe in temperate rice areas [31]. Breeding for CT in rice has always emphasized the evaluation of existing germplasm materials for their ability to withstand low temperatures, aiming to utilize those with strong CT for hybridization and ultimately develop new varieties that are tolerant to low temperatures. While this approach allows for more focused breeding efforts, it also limits the scope of available materials for CT breeding, resulting in a potential issue of limited genetic diversity in subsequent breeding work. The present study extensively evaluated the performance of CT at the seedling stage for 1992 accessions selected from the 3K-RGP, thereby providing a valuable dataset for identifying favorable alleles associated with these cloned CT genes. The identification of numerous accessions exhibiting extreme CT levels will significantly enhance genetic diversity and contribute to future rice breeding efforts targeting CT traits.

The difference of CT between the subspecies may be caused by the temperature difference of latitude and elevation and ecological habitats in different regions [32]. In this study, we observed differential responses to cold stress among various rice subgroups at the seedling stage. The *geng* subspecies exhibited higher CT compared to the *xian* subspecies; however, within the *geng* subgroup, there were variations in CT (Figure 1b,d). Notably, GJ-tmp from China and Europe displayed the highest level of CT with an average survival rate of 78.69% (Figure 1b,d). GJ-adm represented an intermediate type within the *geng* subspecies with a stronger CT (average survival rate: 69.64%). Moreover, GJ-trp from Southeast Asian island countries demonstrated better CT (58.68%) than GJ-sbtrp from Southeast Asian countries (44.97%) (Figure 1b,d). Similarly, in the *xian* subspecies, XI-A (44.97% for SR) from East Asia exhibited greater CT compared to other *xian* subspecies, namely XI-1B, XI-2, XI-3, and XI-adm (ranging from 10.10% to 16.29%). Notably, even within CT-sensitive *xian* subspecies, certain extreme CT accessions were identified such as Ha Goo, Bu Zhi Ming, Ai Da, and Laozaogu of XI-1A with each displaying an SR of 100% from China, Taichun Sen Yu 214 of XI-1B with an SR of 100% from Taiwan, NCS 458 and Derawa of XI-2 with SRs of 97.62% and 96.46%, respectively, from India and Nepal, Gaset Bow of XI-3 with an SR of 94.72% from Nepal, and Padi Ladang Ase Polo Komek and ARC 10581 of XI-*adm* with SRs of 97.87% and 97.98%, respectively, from Indonesia and India. Therefore, to enhance the genetic diversity in rice breeding programs, it is strongly recommended to incorporate diverse CT varieties from *xian* subspecies as donor parents, alongside the utilization of traditional CT-landrace and temperate *geng* varieties. Furthermore, previous research studies [33,34] have demonstrated the occurrence of transgressive performance in abiotic stress tolerance, including CT, surpassing that of the parental lines in a majority of BC populations for almost all abiotic stresses. It is noteworthy that identifying BC progeny with exceptional tolerances was a common observation [33,34]. Therefore, in rice CT breeding practices, it is feasible to incorporate more diverse varieties as donor parents irrespective of their individual performance when combined with stringent selection criteria. This approach will likely result in the broadest genetic variations in CT.

CT is a quantitative trait controlled by complex genetic networks in rice. GWAS is a technique for examining how phenotypic variation and genome-wide genetic variation are generally associated. As a result, GWAS analysis is currently a widely used tool for identifying candidate genes related to complex traits. A total of 16 loci associated with CT at the seedling stage were identified using the whole populations. By comparing the previously reported cloned genes for CT with the mapping results in this study, *qSR1.2a* and *qSCT1.1a* were located on chromosome 1 at 11.71–11.97 Mb and co-localize with the cold-tolerant gene *OsLEA9* [27]. The over-expression of *OsLEA9* significantly decreased the CT of rice during reproductive growth, and the CT of *OsLEA9* knockout lines was significantly stronger than that of the control line [27]. The QTL *qSR1.1x* and *qSCT1.1x* were located on chromosome 1 at 3.45–3.75 Mb and co-localize with the cold-tolerant gene *OsPLDα1*, which played an important role in cold signal transduction in rice by producing phosphatidic acid (PA) and regulated the expression of *OsDREB1* via *OsMPK6*, *OsSIZ1*, and other PA-binding proteins [29]. The QTL *qSCT2.1a* was found in the 28.66–28.96 Mb of chromosome 2 and co-located with the transcription factor *LGS1*, which influenced grain size and could improve rice seedling CT and survival following cold stress treatment [28]. We detected some QTLs that had been previously located, indicating the accuracy of the mapping results by GWAS in CT-related traits in this study.

Bioinformatics was used to further infer candidate genes. We used MBKBASE’s RNA-seq database to assess the expression patterns of candidate genes for each QTL to find potential candidate genes (Figure S3). Among the candidate gene, *OsELF4a* (*LOC_Os11g40610*) for *qSR11.1x* combined with *OsELF3-1* and *OsLUX* to constitute a terpolymer inhibitor complex *OsEC1*, which played a role in heading and stress tolerance [35]. Under a short day, the heading time of mutant *oself4a* was delayed for 8 days, and all internodes were shortened, with semi-dwarf, short ear, grain length, grain width, and grain weight all decreased [30]. The survival rate and setting rate of *oself4a* decreased under salt stress [30]. So, *LOC_Os11g40610* (Figure S3) was inferred as a most likely candidate gene affecting CT in rice. Another candidate gene of *qSR3.1a*, *LOC_Os03g44760*, which encoded a protein with a coiled-helix domain in an intermediate region, was expressed at the seedling, heading, grain milk, and endosperm filling stages, particularly in the ovules at the heading stage (Figure S4), and its expression level increased when exposed to abiotic stress (http://ipf.sustech.edu.cn/pub/ricerna/, accessed on 30 August 2024). The *LOC_Os03g44760* gene was homologous to *SWI1* in Arabidopsis and *AM1* in maize, which was mostly found in prophase I of sex blast cells, and was essential for the development of the proper chromosomal shape during meiosis commencement [36]. However, the meiosis process was very vulnerable to environmental influences, particularly high and low temperature stress, resulting in pollen abortion and decreased seed setting rate [37,38]. *LOC_Os03g44760* was the most likely candidate gene and worthy of further verification by a gene-editing or transgenic approach. To identify the CT of the above candidate genes, we need to create transgenic materials to verify gene function.

To date, several CT genes have been cloned in rice, such as *COLD1*, *bZIP73*, and *qPSR10*, which enhance CT in cultivated rice derived from wild relatives [39,40,41]. Additionally, *CTB4a* and *Ctb1* have been retained during the adaptation to cold climate conditions in temperate cultivars [2,6], while *HAN1* has undergone mutations in temperate cultivars for improved cold adaptation [5]. However, it appears that these cloned CT genes have not yet found widespread utilization in rice breeding programs focused on enhancing CT or are already present in modern cultivars. Therefore, the identification of novel CT genes from germplasm resources remains a crucial task. Based on haplotype analysis of the important candidate genes identified in this study, the pyramiding of *LOC_Os03g44760*^Hap3^ at *qSR3.1a/qSCT3.1a*, *LOC_Os04g07260*^Hap3^ at *qSR4.1a*/*qSCT4.1a*/*qSR4.1x*/*qSCT4.1x*, *LOC_Os11g40610*^Hap3^ at *qSR11.1x*/*qSCT11.1x*, and *LOC_Os12g10710*^Hap2^ at *qSR12.1a*/*qSR12.1x*/*qSCT12.1x* could significantly enhance CT at the rice seedling stage. Three accessions CX534, B236, and IRIS_313-8565 were identified, each having an SR of 100% and an SCT of 1. CX534 from China carries four favorable haplotypes (*LOC_Os03g44760*^Hap3^, *LOC_Os04g07260*^Hap3^, *LOC_Os11g40610*^Hap3^, and *LOC_Os12g10710*^Hap2^) with a middle-late ripening cultivar with a flat blade leaf, oval grain type, red glume coloration along with glume tip. B236 from China carries three favorable haplotypes (*LOC_Os04g07260*^Hap3^, *LOC_Os11g40610*^Hap3^, and *LOC_Os12g10710*^Hap2^) with high alkali tolerance, fertilizer use efficiency, semi-dwarf, and high seed setting rate; it also exhibits low temperature tolerance at the booting stage. IRIS_313-8565 from Thailand harbors two favorable haplotypes (*LOC_Os03g44760*^Hap3^ and *LOC_Os12g10710*^Hap2^) with semi-dwarf, medium leaf size, high grain quality, and lodging resistance. Our findings demonstrate that rice germplasms carrying more favorable haplotypes for candidate genes exhibit improved CT (Figure 6), suggesting that developing an optimal combination of haplotypes through pyramiding multiple favorable alleles can be an effective strategy to enhance CT at the seedling stage. Therefore, the above three accessions (CX534, B236, and IRIS_313-8565) can serve as donor parents in rice breeding of CT through the introgression of diverse favorable alleles into elite varieties that are susceptible to cold stress by MAS.

## 5. Conclusions

A phenotypic analysis of 1992 rice materials showed that *geng* accessions were more cold-tolerant than *xian* accessions and the *GJ-tmp* subgroup had the strongest CT at the seedling stage. A total of 29 QTLs were identified as associated with CT at the seedling stage on the basis of two traits (SR and SCT) by GWAS analyses. Five candidate genes (*LOC_Os03g44760*, *LOC_Os04g06900*, *LOC_Os04g07260*, *LOC_Os11g40610*, and *LOC_Os12g10710*) were identified through gene function annotation and haplotype analysis. Our findings indicate that rice germplasms pyramiding more favorable haplotypes for candidate genes exhibit improved CT. Then, three accessions (CX534, B236, and IRIS_313-8565) carried different superior alleles that were identified for use in the molecular breeding of CT in rice through marker-assisted selection (MAS). The result of this study provided resources for improving rice CT and laid the groundwork for the future cloning of new CT genes.

## Figures and Tables

**Table 1:** QTLs identified for two cold-tolerance traits by GWAS in the whole population.

Trait ^a^	QTL	Chr.	QTL Region (Mb)	Lead SNP	*p*-Value	Cloned Gene
SR	*qSR1.1a*	1	6.37–6.67	6,518,020	6.62 × 10^−8^	
	*qSR1.2a*	1	11.71–12.01	11,864,873	6.05 × 10^−7^	*OsLEA9* [27]
	*qSR1.3a*	1	19.98–20.27	20,125,327	6.39 × 10^−7^	
	*qSR2.1a*	2	6.26–6.56	6,416,777	3.57 × 10^−7^	
	*qSR3.1a*	3	25.10–25.55	25,249,852	1.36 × 10^−7^	
	*qSR4.1a*	4	3.58–3.96	3,633,378	2.10 × 10^−10^	
	*qSR5.1a*	5	19.63–19.93	19,779,633	5.72 × 10^−9^	
	*qSR12.1a*	12	5.60–5.90	5,753,724	5.54 × 10^−8^	
SCT	*qSCT1.1a*	1	11.53–11.97	11,956,876	4.74 × 10^−8^	*OsLEA9* [27]
	*qSCT1.2a*	1	28.47–28.77	28,623,028	1.88 × 10^−7^	
	*qSCT2.1a*	2	28.66–28.96	28,812,677	4.12 × 10^−7^	*LGS1* [28]
	*qSCT3.1a*	3	25.11–25.41	25,260,301	8.78 × 10^−7^	
	*qSCT4.1a*	4	3.48–3.78	3,633,378	5.60 × 10^−8^	
	*qSCT8.1a*	8	9.18–9.48	9,334,380	8.64 × 10^−7^	
	*qSCT8.2a*	8	12.60–12.90	12,755,109	1.24 × 10^−7^	
	*qSCT9.1a*	9	14.24–14.54	14,393,189	5.25 × 10^−7^	

^a^ SR—survival rate, SCT—leaf score of cold tolerance.

**Table 2:** QTLs identified for the two cold-tolerance traits by GWAS in the *geng* (GJ) and *xian* (XI) subgroups.

Population	Trait ^a^	QTL	Chr.	QTL Region (Mb)	Lead SNP	*p*-Value	Cloned Gene
GJ	SR	*qSR2.1g*	2	19.64–19.94	19,785,654	4.94 × 10^−6^	
XI	SR	*qSR1.1x*	1	3.45–3.75	3,604,713	4.19 × 10^−7^	*OsPLDα1* [29]
		*qSR1.2x*	1	20.23–21.03	20,231,449	1.39 × 10^−7^	
		*qSR4.1x*	4	3.63–4.80	3,855,187	3.33 × 10^−10^	
		*qSR5.1x*	5	5.71–6.01	5,861,711	1.21 × 10^−6^	
		*qSR11.1x*	11	23.79–25.42	25,393,345	8.00 × 10^−9^	
		*qSR12.1x*	12	5.71–7.25	5,754,489	6.05 × 10^−11^	
XI	SCT	*qSCT1.1x*	1	3.45–3.75	3,604,730	1.34 × 10^−7^	*OsPLDα1* [29]
		*qSCT1.2x*	1	21.88–22.18	22,038,470	9.39 × 10^−7^	
		*qSCT4.1x*	4	3.50–3.80	3,653,716	1.19 × 10^−7^	
		*qSCT8.1x*	8	1.42–1.72	1,578,048	5.94 × 10^−7^	
		*qSCT11.1x*	11	24.03–24.33	25,393,345	3.17 × 10^−7^	
		*qSCT12.1x*	12	5.71–7.33	5,754,489	4.48 × 10^−7^	

^a^ SR—survival rate, SCT—leaf score of cold tolerance.

## Data Availability

The datasets supporting the conclusions of this article are included within the article and Supplementary Materials.

## Supplementary Materials

The following supporting information can be downloaded at: https://www.mdpi.com/article/10.3390/biology13100784/s1, Figure S1: the linkage distance (LD) of 1992 samples used in this study. Figure S2: Frequency distributions of survival rate (SR) and leaf score for cold tolerance (SCT) in the whole population (a and b), as well as in the *geng* (c and d) and *xian* (e and f) subpopulations. Figure S3: Expression pattern of candidate gene of *LOC_Os11g40610*. Figure S4: Expression pattern of candidate gene of *LOC_Os03g44760*. Table S1: The 1992 rice accessions used in this study and their cold tolerance measured at the seedling stage.

## Abbreviations

3K-RGP: 3K Rice Genome Project; CT: cold tolerance; GJ: *geng*; XI: *xian*.
