# Supplementary material for: Identification of Candidate Genes for Cold Tolerance at Seedling Stage by GWAS in Rice (Oryza sativa L.)
Source: Biology (Basel). 2024 Sep 30;13(10):784. doi: 10.3390/biology13100784 (PMC11505075; doi:10.3390/biology13100784)
Supplement: Supplementary file 1 [file biology-13-00784-s001.zip › Figure S2.pptx]

## Slide 1
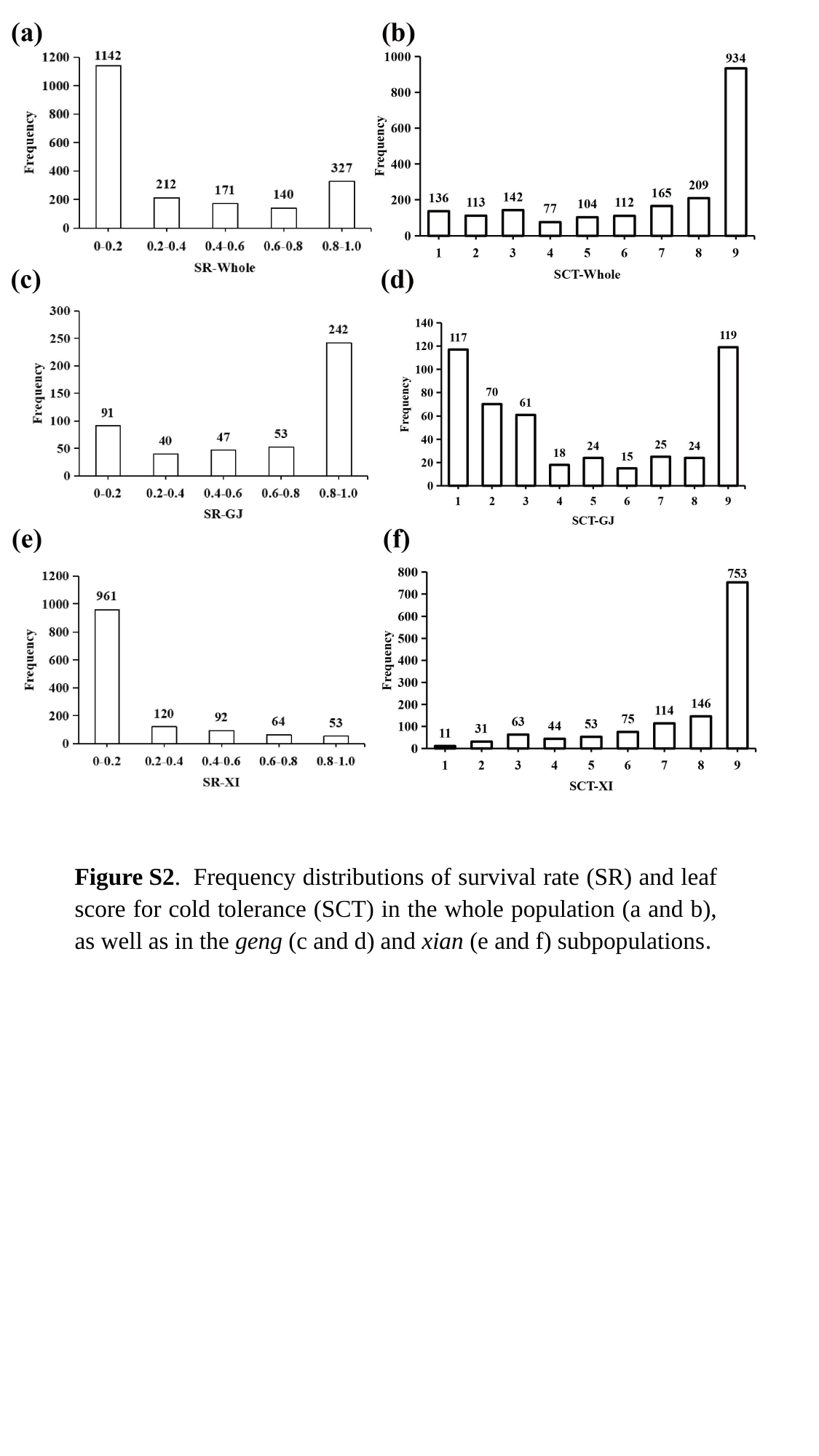

Figure S2.  Frequency distributions of survival rate (SR) and leaf score for cold tolerance (SCT) in the whole population (a and b), as well as in the geng (c and d) and xian (e and f) subpopulations.
