# Supplementary material for: Identification of Candidate Genes for Cold Tolerance at Seedling Stage by GWAS in Rice (Oryza sativa L.)
Source: Biology (Basel). 2024 Sep 30;13(10):784. doi: 10.3390/biology13100784 (PMC11505075; doi:10.3390/biology13100784)
Supplement: Supplementary file 1 [file biology-13-00784-s001.zip › Figure S3.pptx]

## Slide 1
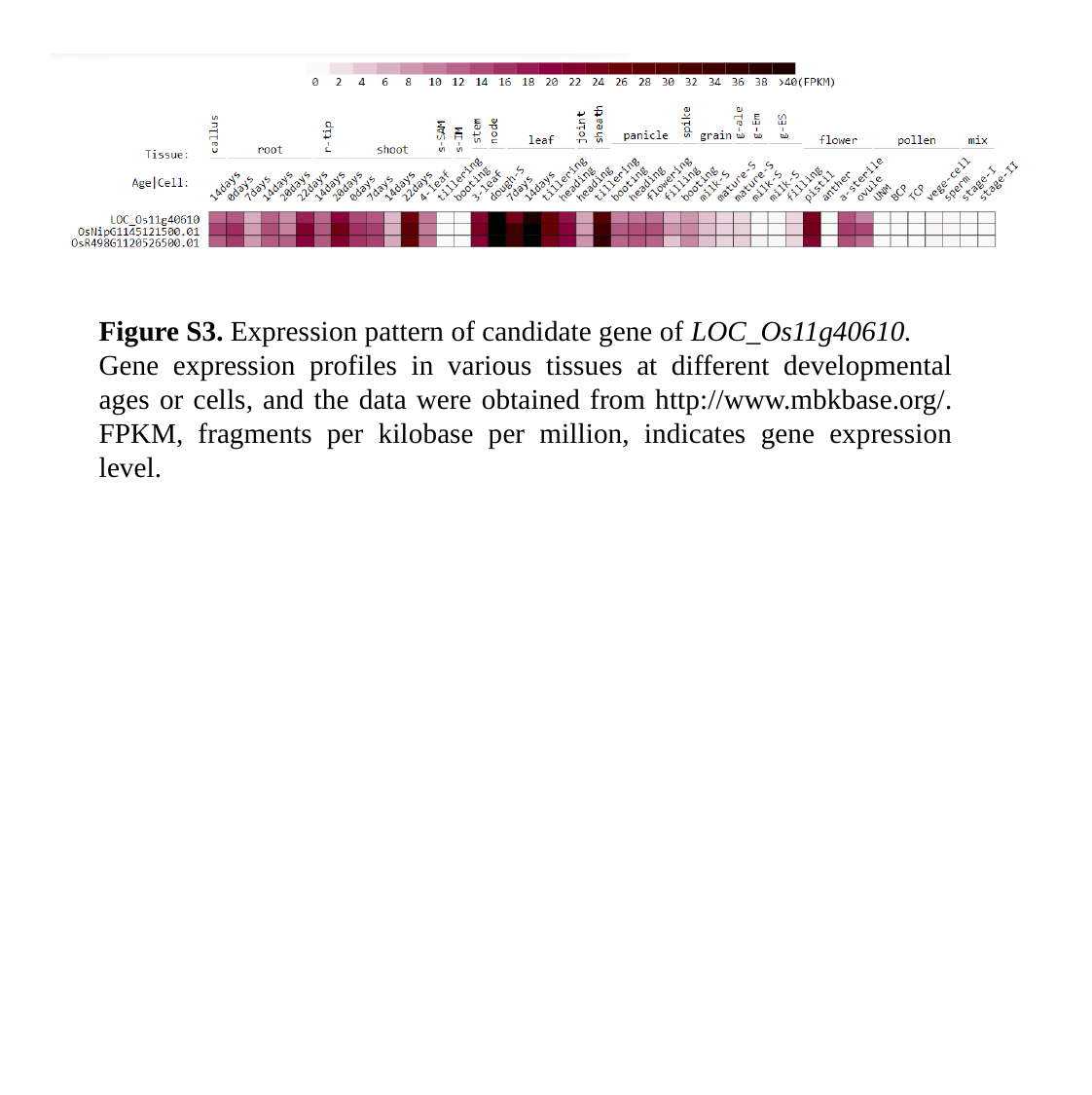

Figure S3. Expression pattern of candidate gene of LOC_Os11g40610.
Gene expression profiles in various tissues at different developmental ages or cells, and the data were obtained from http://www.mbkbase.org/. FPKM, fragments per kilobase per million, indicates gene expression level.
