# Supplementary figures and images for: Identification of Candidate Genes for Cold Tolerance at Seedling Stage by GWAS in Rice (Oryza sativa L.)
Source: Biology (Basel). 2024 Sep 30;13(10):784. doi: 10.3390/biology13100784 (PMC11505075; doi:10.3390/biology13100784)

## Slide 1
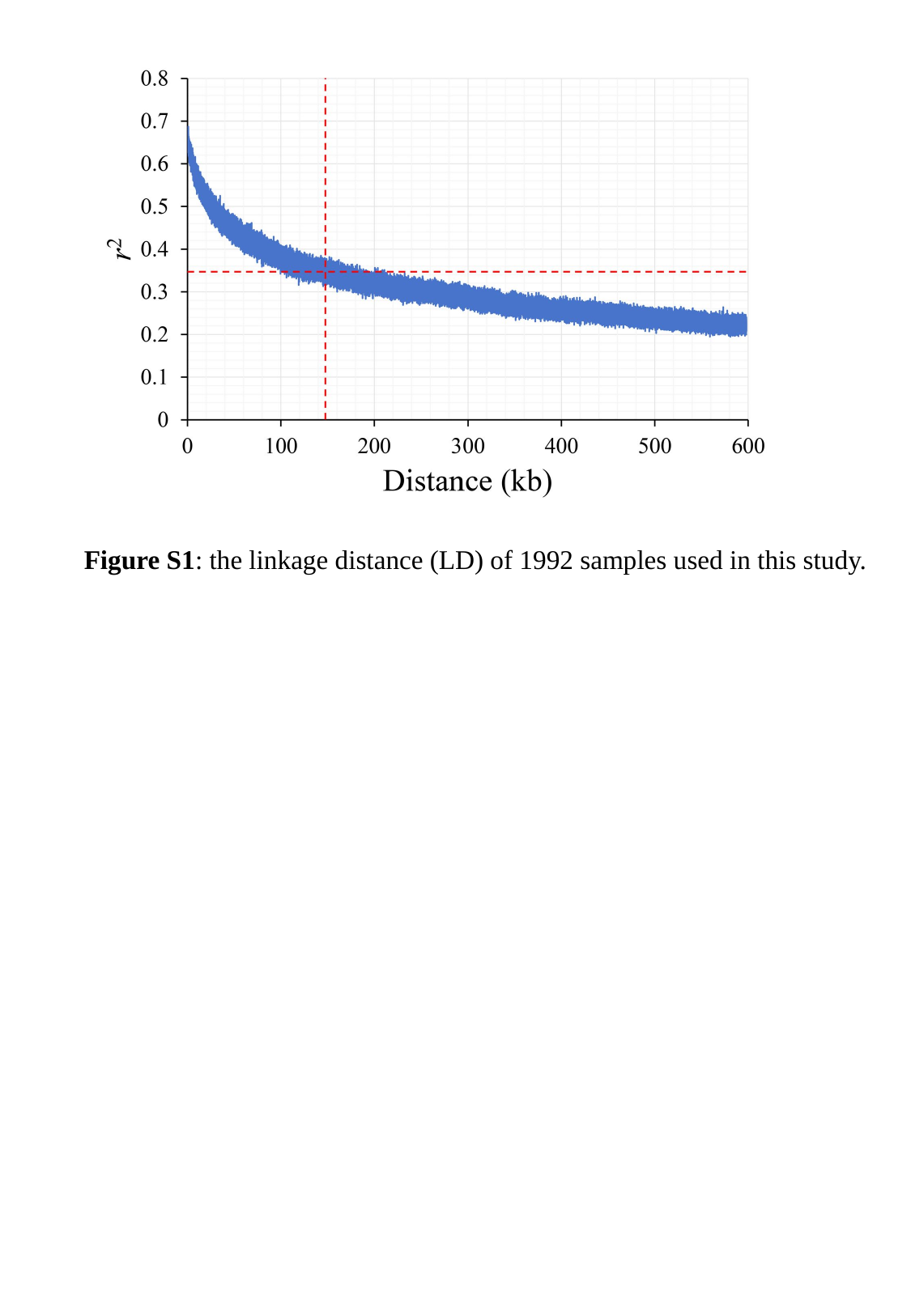

Figure S1: the linkage distance (LD) of 1992 samples used in this study.

Supplement: Supplementary file 1 [file biology-13-00784-s001.zip › Figure S1.pptx]
